# Trends in Utilization of Guideline-Directed Cardiorenal Protective Therapies for Chronic Kidney Disease in Patients with Cardiovascular Morbidity: Real World Data from Two Cross-Sectional Snapshots (HECMOS I and II)

**DOI:** 10.3390/biomedicines13081987

**Published:** 2025-08-15

**Authors:** Panagiotis Theofilis, Ioannis Leontsinis, Dimitrios Farmakis, Dimitrios Avramidis, Nikolaos Argyriou, Matthaios Didagelos, Ioannis Zarifis, Costas Thomopoulos, Anastasia Kitsiou, Georgios Koutsopoulos, George Kourgianidis, Athanasios Kostopoulos, Eleni Manta, Maria Marketou, Vasiliki Bistola, George Bibis, Katerina K. Naka, Periklis Ntavlouros, Evangelos Oikonomou, Sotirios Patsilinakos, Nikolaos Patsourakos, Asaf Sawafta, Vaios Schismenos, Athanasios Trikas, Georgios Chalikias, Christos Chatzieleftheriou, Konstantinos Tsioufis

**Affiliations:** 1 1st Cardiology Department, “Hippokration” General Hospital of Athens, University of Athens Medical School, 11527 Athens, Greece; panos.theofilis@hotmail.com (P.T.); nikos_ar@hotmail.com (N.A.); giorgoskoutsopoulos93@gmail.com (G.K.); elenmanta@gmail.com (E.M.); ktsioufis@gmail.com (K.T.); 2 2nd Cardiology Department, “Attikon” University Hospital, University of Athens Medical School, 11527 Athens, Greece; dimitrios_farmakis@yahoo.com (D.F.); vasobistola@yahoo.com (V.B.); 3 Cardiology Department, “G. Gennimatas” General Hospital, 11527 Athens, Greece; d_avramides@yahoo.com; 4 1st Cardiology Department, AHEPA University General Hospital, 54636 Thessaloniki, Greece; manthosdid@yahoo.gr; 5 Cardiology Department, “G. Papanicolaou” Hospital, 57010 Thessaloniki, Greece; zarifis.john@gmail.com; 6 Department of Cardiology, General Hospital of Athens “Laiko”, 11527 Athens, Greece; thokos@otenet.gr; 7 Cardiology Department, Sismanoglio General Hospital, 15126 Athens, Greece; anastasia.kitsiou@gmail.com; 8 Cardiology Department, 251 Air Force General Hospital, 11525 Athens, Greece; kourgiannidis.medicalring@gmail.com; 9 1st Cardiology Department, General Hospital of Nikea-Piraeus, 18454 Nikea Agios Ioannis Rentis, Greece; kgkostop@gmail.com; 10 Cardiology Department, PAGNI University Hospital, 71500 Heraklion, Greece; marymarke@yahoo.gr; 11 Cardiology Department, General Hospital of Argos, 21200 Argos, Greece; georbibis@yahoo.gr; 12 Cardiology Department, Ioannina University Hospital, 45500 Ioannina, Greece; anaka@uoi.gr; 13 Cardiology Department, Patras University Hospital, 26504 Rio, Greece; pdav@upatras.gr; 14 3rd Cardiology Department, “Sotiria” Chest Diseases Hospital, University of Athens Medical School, 11527 Athens, Greece; boikono@gmail.com; 15 Cardiology Department, Konstantopoulio General Hospital, 14233 Athens, Greece; spatsilinakos@gmail.com; 16 Cardiology Department, “Tzaneio” General Hospital of Piraeus, 18536 Piraeus, Greece; drpats@yahoo.gr; 17 Department of Cardiology, University Hospital of Larissa, 41334 Larissa, Greece; assaf.sawafta91@hotmail.com; 18 Cardiology Department, “Hippokration” General Hospital of Thessaloniki, 54636 Thessaloniki, Greece; vaiosschism@gmail.com; 19 Cardiology Department, Evangelismos General Hospital, 11525 Athens, Greece; atrikas@otenet.gr; 20 Cardiology Department, Alexandroupolis University Hospital, 68100 Alexandroupolis, Greece; gchaliki@med.duth.gr; 21 Cardiology Department, Drama General Hospital, 66100 Drama, Greece; hatzih62@gmail.com

**Keywords:** chronic kidney disease, finerenone, heart failure, SGLT2 inhibitor

## Abstract

**Introduction:** Chronic kidney disease (CKD) affects roughly 10% of the global population and significantly increases cardiovascular risk. While renin–angiotensin system inhibitors (RASi) remain a therapeutic mainstay, recent evidence supports the renoprotective value of sodium–glucose cotransporter-2 inhibitors (SGLT2i) and finerenone. This study evaluated the real-world use of guideline-directed medical therapy (GDMT) among patients with cardiorenal disease in Greece and explored factors influencing prescribing patterns. **Methods:** The Hellenic Cardiorenal Morbidity Snapshots (HECMOS 1 and 2) enrolled all cardiology inpatients across Greece on 3 March, 2022, and 5 June, 2024. Comorbidities and medication data were based on self-report and chart review. CKD patients eligible for SGLT2i and finerenone were identified per guideline criteria. Multivariable logistic regression was used to identify predictors of SGLT2i use. **Results:** From a total of 923 and 1222 patients enrolled in HECMOS 1 and 2, CKD was present in 26% and 27%, respectively. SGLT2i use prior to hospitalization rose from 15% in HECMOS 1 to 30.4% in HECMOS 2. In HECMOS 1, diabetes mellitus was the strongest predictor of SGLT2i use (OR 12.01, 95% CI 3.31–45.56, *p* < 0.001), while heart failure predicted use in HECMOS 2 (OR 4.10, 95% CI 1.70–9.88, *p* = 0.002). Finerenone was prescribed in only 1.7% of eligible patients in HECMOS 2. RASi usage among CKD patients remained stable across both cohorts (42.1% vs. 41.7%), with renal dysfunction showing no impact on prescribing patterns. **Conclusions:** SGLT2i use in patients with CKD and cardiovascular disease doubled over 2 years, indicating progress in implementing GDMT. However, overall use of disease-modifying therapies remains suboptimal, underscoring the need for further improvement in real-world care.

## 1. Introduction

Chronic kidney disease (CKD) is a major global health burden, affecting approximately 10% of the world’s population and contributing to significant morbidity, mortality, and healthcare costs [1]. Characterized by a gradual decline in kidney function, CKD often progresses to end-stage renal disease (ESRD) if left untreated, necessitating dialysis or kidney transplantation. Beyond its direct impact on renal health, CKD is closely associated with extrarenal complications, namely cardiometabolic disease. Early diagnosis and effective treatment are crucial to decelerate disease progression, enhance patient outcomes, and alleviate the associated with advanced CKD economic and societal costs. Consequently, developing and implementing guidelines recommending renoprotective therapies [2] are strategies of utmost importance.

Until recently, the only available effective pharmaceutical treatment in slowing the progression of kidney function decline was renin–angiotensin system inhibition (RASi), either through angiotensin-converting enzyme inhibitors or angiotensin II receptor blockers. However, recent clinical trial data (CREDENCE, DAPA-CKD, EMPA-KIDNEY) established the sodium–glucose cotransporter-2 inhibitors (SGLT2i) to be not only safe in patients with impaired kidney function but also efficient with regards to improved renal and cardiovascular outcomes [3]. Along with heart failure (HF) [4], these are now offered as standard of care in CKD. Furthermore, finerenone, a non-steroidal mineralocorticoid receptor antagonist (MRA) that decelerated CKD progression and lowered the incidence of adverse cardiovascular outcomes in patients with type 2 diabetes mellitus (T2DM) and CKD [5,6], represents the third pillar of the current renoprotective triad in these patients [7].

Given the well-documented benefit of the aforementioned novel renoprotective agents, we sought to describe the uptake and predictors of guideline-directed medical therapy (GDMT) in Greek cardiology inpatients with CKD at two time points.

## 2. Methods

### 2.1. Study Population

Hellenic Cardiorenal Morbidity Snapshot (HECMOS) 1 [8] and HECMOS 2 were two independent, cross-sectional snapshots conducted at different time points in 55 and 71 cardiology departments across Greece, respectively. They aimed to evaluate various aspects of cardiorenal morbidity, including the real-world prescription patterns of renoprotective therapies, such as SGLT2i and finerenone, in patients with CKD. Data collection, including both demographic and clinical characteristics of all participants, occurred on 2 March 2022, for HECMOS 1. HECMOS 2 occurred on 5 June 2024.

CKD in the HECMOS was defined as an estimated glomerular filtration rate (eGFR) equal to or less than 60 mL/min/1.73 m^2^ over the last 3 months or albuminuria satisfying the Kidney Disease Improving Global Outcomes (KDIGO) CKD diagnostic criteria (>30 mg/g) [7]. CKD patients eligible for RASi, SGLT2i, and finerenone therapy were identified according to accepted criteria [7], which include those with an eGFR of ≥25 mL/min/1.73 m^2^. It should be noted that finerenone was not available in Greece during HECMOS 1. Patients with advanced CKD (eGFR < 25 mL/min/1.73 m^2^) and those requiring renal replacement therapy were excluded from certain analyses.

### 2.2. Variables

The study variables included demographic data (age, sex, BMI), comorbidities (diabetes mellitus, chronic HF (CHF), atherosclerotic cardiovascular disease), prior CKD-related hospitalizations, and whether patients had previously been reviewed by a nephrologist. Medication history captured prior use of RASi, SGLT2i, diuretics, MRAs, and finerenone. SGLT2i and finerenone prescriptions prior to hospital admission were analyzed for eligibility criteria compliance and predictor association. Data were collected through medical records and patient self-reports at the time of admission. Εach recruiter confirmed drug history through prescription records for each patient.

### 2.3. Statistical Analysis

Continuous variables are expressed as mean (standard deviation), while categorical variables are reported as counts and percentages. For comparisons of continuous variables, the two-sample t-test or Mann-Whitney u test was used, as appropriate, and differences in categorical variables were assessed with the Chi-squared test. These statistical analyses were performed using SPSS version 23.0 (SPSS Inc., Chicago, IL, USA), with a two-sided *p*-value of less than 0.05 considered statistically significant. We also performed a multilevel logistic regression using the glmer function from the lme4 package in R studio (version 2025.05.1+513) to identify predictors of SGLT2i use. The variables included were chosen according to a univariate *p*-value < 0.10, and hospital ID was included as a random intercept to account for institutional clustering. Intraclass correlation coefficients (ICCs) were calculated to assess the proportion of variance explained by hospital-level differences.

## 3. Results

### 3.1. Characteristics of the Study Population

**Figure 1:**
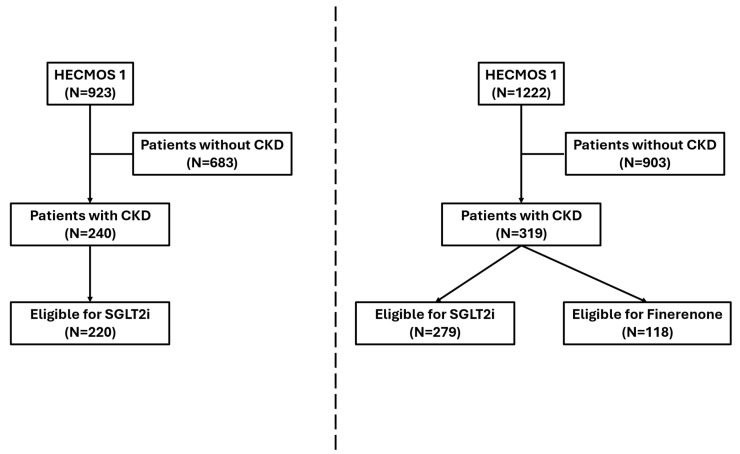
Flowchart of patient selection in HECMOS 1 and 2. CKD: chronic kidney disease, SGLT2i: sodium-glucose cotransporter-2 inhibitor.

A total of 923 patients hospitalized in cardiology departments of 55 hospitals across Greece on 3 March 2022, and 1222 patients hospitalized in cardiology departments of 71 hospitals across Greece on 5 June 2024, were enrolled in HECMOS 1 and 2, respectively, and were included in this analysis (Figure 1). Among them, 240 (26.4%) patients and 319 (26.1%) patients had a history of CKD in HECMOS 1 and 2, respectively. CKD patients were older, with a remarkably higher prevalence of comorbidities such as hypertension, DM, atrial fibrillation, atherosclerotic cardiovascular disease, and CHF compared to patients without CKD, in both cohorts (Supplementary Table S1). Of note, arterial hypertension (HECMOS 1: 76.7%, HECMOS 2: 79.1%) and DM (HECMOS 1: 54.4%, HECMOS 2: 45.8%) were highly prevalent comorbidities in the CKD population. Regarding medical therapy prior to the index hospitalization, which was verified in 100% of participants through prescription records, those with a history of CKD more often received diuretics, MRAs, and SGLT2i. Interestingly, RASi was prescribed at similar rates in CKD and non-CKD patients (HECMOS 1: CKD: 41.7% vs. non-CKD: 43.7%, *p* = 0.54; HECMOS 2: CKD: 42.1% vs. non-CKD: 44.8%, *p* = 0.47). ESRD on chronic renal replacement therapy was reported in 7.6% and 10.9% of the CKD patients in HECMOS 1 and 2, respectively. Less than half of the CKD patients were under outpatient nephrology consultation.

### 3.2. GDMT Use in Patients with CKD

**Figure 2:**
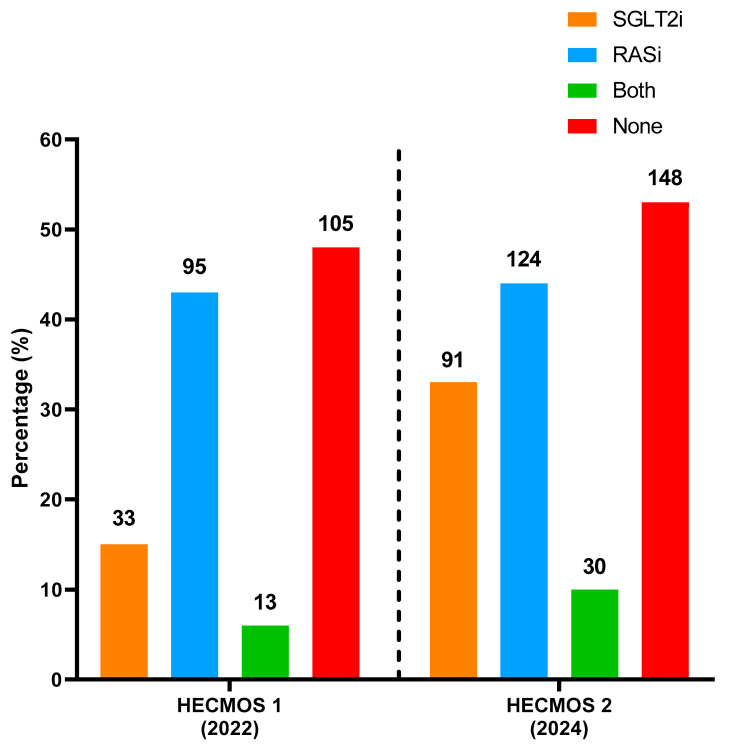
Guideline-directed medical therapy prescription prior to admission in CKD HECMOS. SGLT2i: sodium–glucose cotransporter-2 inhibitor, RASi: renin-angiotensin system inhibitor. The exact number of patients is shown above the bars.

After excluding patients with advanced CKD, 220 and 279 individuals in HECMOS 1 and 2 were analyzed regarding the prescription status of GDMT prior to the index hospitalization. The utilization of GDMT in CKD patients in HECMOS is shown in Figure 2. Although SGLT2i prescription rates demonstrated a substantial increase, approximately 50% of the population did not receive any reno-protective GDMT.

The characteristics of SGLT2i users and non-users are presented in Supplementary Table S2. In HECMOS 1, 15% of the CKD population were on SGLT2i. Compared to non-users, SGLT2i users were younger (73.7 (10.3) years vs. 78.0 (10.0) years, *p* = 0.03), and the vast majority had DM (90.6% vs. 47.5%, *p* < 0.001). At the same time, they commonly had a history of CHF (93.8% vs. 73.1%, *p* = 0.01) and ASCVD (69.7 vs. 49.2, *p* = 0.03). Age, history of CHF, and the presence of DM were associated with SGLT2i use based on the multivariable regression analysis (Supplementary Table S3).

In HECMOS 2, approximately one-third of patients eligible for SGLT2i treatment were on this drug class. SGLT2i users were younger (76.5 (9.6) years vs. 80.0 (9.9) years, *p* = 0.008) and more frequently male (74.2% vs. 61.3%, *p* = 0.04) compared to non-users. Moreover, SGLT2i users were predominantly patients with coexisting CHF and were receiving diuretics and MRAs at a higher frequency. However, RASi (including angiotensin receptor neprilysin inhibitors, ARNI) was similarly prescribed in SGLT2i users and non-users (51.6% vs. 52.1%, *p* = 0.94). Interestingly, consultation with a nephrologist did not appear to influence SGLT2i prescription rates in this group of patients (consultation: 32.3% vs. no consultation: 33.2%, *p* = 0.88).

In HECMOS 1, diabetes was the strongest independent predictor of SGLT2i use (OR: 17.00, *p* < 0.001), followed by chronic heart failure (OR: 5.55, *p* = 0.068) and younger age (OR: 0.93 per year increase, *p* = 0.019). In HECMOS 2, CHF emerged as the primary predictor (OR: 5.23, *p* < 0.001), while diabetes (OR: 1.38, *p* < 0.001) and vascular disease (OR: 1.73, *p* = 0.049) were also associated with increased likelihood of SGLT2i use. Age remained a negative predictor (OR: 0.97 per year, *p* < 0.001). The ICC was 37.3% and 16.4% for HECMOS 1 and 2, respectively, indicating substantial inter-hospital variation.

**Figure 3:**
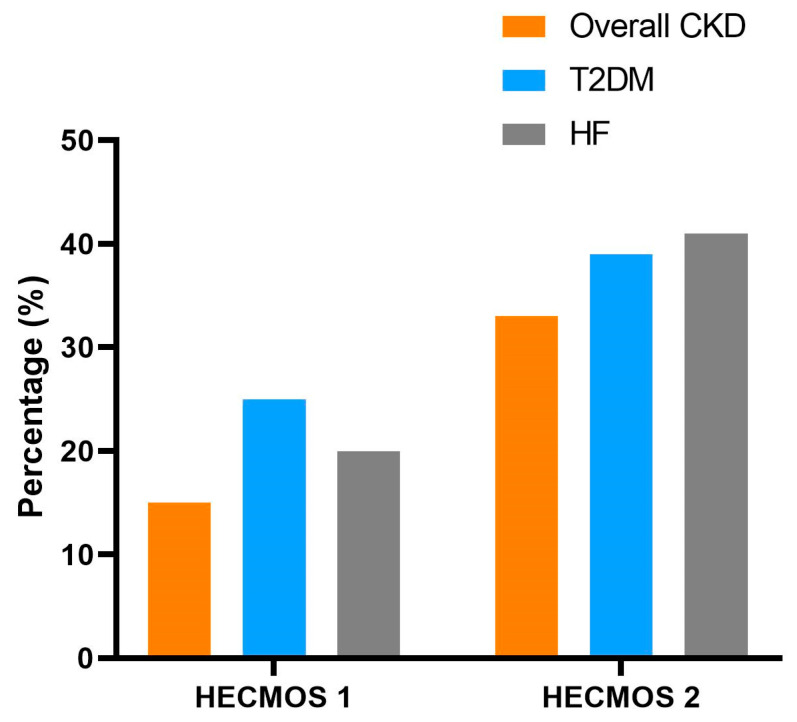
SGLT2i prescription in overall chronic kidney disease (CKD), CKD with type 2 diabetes mellitus (T2DM), and CKD with heart failure (HF).

Considering the different SGLT2i indications, their utilization rates with regards to the presence of CKD with either DM or HF are shown in Figure 3. Finally, at the time of the HECMOS 2, 132 CKD patients had eGFR values of ≥20 mL/min/1.73 m^2^ and had not previously received SGLT2i treatment. Yet only 26 of these patients (19.7%) had initiated SGLT2i therapy.

After further exclusion of patients without T2DM and those with a history of hyperkalemia, we analyzed 118 cases eligible for treatment with finerenone in HECMOS 2. Prior to hospitalization, only two patients (1.7%) were receiving finerenone, while none of the eligible patients had initiated finerenone at the time of the snapshot. Concerning patients with a history of hyperkalemia, only one (4%) was on a potassium binder.

## 4. Discussion

This analysis of HECMOS highlights a notable gap between guideline-directed recommendations for renoprotective therapies in CKD and their real-world implementation in clinical practice regarding patients with cardiovascular morbidity. SGLT2i use was reported in 15% of CKD patients in HECMOS 1, predominantly in patients with a history of DM and HF, as these were their only established indications at that time. In HECMOS 2, 32.6% of eligible CKD patients were prescribed SGLT2i, and only 1.7% were receiving finerenone. These results highlight a substantial improvement in incorporating available evidence and guideline recommendations for SGLT2i in clinical practice during the last 2 years. Nevertheless, there is definitely room for improvement.

The prescribing patterns observed in HECMOS 1 and 2 should be interpreted in the context of evolving guideline recommendations and the timing of national reimbursement in Greece. At the time of HECMOS 1 (May 2022), SGLT2 inhibitors were already included in European Society of Cardiology (ESC) guidelines [9] as a foundational therapy for patients with HF and a reduced ejection fraction (HFrEF). However, their reimbursement in Greece was initially limited to type 2 diabetes and, as of April 2022, HFrEF. It was not until February 2023—between HECMOS 1 and HECMOS 2—that reimbursement was expanded to cover CKD without diabetes. Furthermore, broader HF indications were added in late 2023 [4] prior to HECMOS 2. Therefore, the modest uptake seen in HECMOS 1 may also stem from this evolving landscape. The significant increase observed in HECMOS 2 likely reflects both the expanded reimbursement for CKD and the increasing familiarity among cardiologists with SGLT2i indications due to their prominence in HF guidelines. Importantly, because both snapshots were limited to cardiology departments, the preferential prescribing to patients with CHF may also reflect specialty-driven familiarity rather than nephrology-led practice changes.

Among possible explanations of this slow uptake of SGLT2i and Finerenone is physician ignorance, incredibility, and inertia in adopting these novel renoprotective therapies. This has emerged as a central challenge in contemporary CKD management [10]. In the context of SGLT2i and finerenone, several factors may contribute to inertia, including a lack of familiarity with the latest CKD treatment guidelines, concerns over potential adverse effects (such as euglycemic ketoacidosis with SGLT2i or hyperkalemia with finerenone), and the complexity of managing polypharmacy in CKD patients, who often have multiple comorbidities [10]. Additionally, traditional reliance on established therapies, such as RASi, may overshadow newer treatment options, even when they demonstrate superior outcomes for CKD progression and cardiovascular risk reduction [10]. These inertias may also reflect gaps in training or continuing education, as well as structural barriers within healthcare settings, such as insufficient clinical decision support and limited access to multidisciplinary care that integrates nephrology, cardiology, and primary care perspectives [10].

Interestingly, female sex was associated with lower SGLT2i prescription rates. This observation may be linked to the higher incidence of fungal infections reported among women receiving this therapy. However, our dataset does not differentiate between patients who discontinued SGLT2i treatment and those who were never prescribed it. It is noteworthy that concerns about potential complications often lead physicians to hesitate in initiating treatment. Despite these challenges, it is crucial to emphasize that SGLT2i efficacy is not influenced by gender. Therefore, such barriers should not be allowed to compromise the prognosis of patients with CKD.

Our study’s findings also highlight the role of comorbidities, such as CHF, in influencing the prescription of SGLT2i. Multivariable analysis revealed that a history of CHF was significantly associated with SGLT2i use in both HECMOS, which aligns with current evidence demonstrating cardiovascular benefits of SGLT2i in HF populations [11]. However, a particular trend was evident. While SGLT2i treatment was mainly driven by DM presence in HECMOS 1, the HECMOS 2 results demonstrate a clear shift in Cardiology practice as the SGLT2i were clearly linked with CHF treatment rather than DM. Nevertheless, over half of CKD patients with CHF were not prescribed SGLT2i, indicating that even among patients at high risk, substantial underuse of these therapies persists. A recent study conducted on the Veteran Affairs health system has shown that in over 100,000 patients with HF, T2DM, or ASCVD who may benefit from SGLT2i treatment, only 14.6% were on such therapy. Strikingly, patients with better kidney function were more likely to receive SGLT2i [12]. Additionally, according to a smaller-scale, two-center study in hospitalized patients with HF, SGLT2i was used in less than 5% [13]. These trends underscore the need for better guideline dissemination and a stronger emphasis on their implementation in clinical practice. While underutilization of GDMT in CKD has been observed in other populations, few studies have focused on cardiology inpatients. Our work provides unique real-world insights into this high-risk group.

It is important to highlight that robust beneficial data about SGLT2i and Finerenone treatment in CKD have been available well before the conduction of our study, and also that both these treatments have been commercially available and fully reimbursed in Greece at the time of HECMOS 2. Regarding Finerenone, it became available in the Greek market with reimbursement for diabetic kidney disease in October 2023, prior to the release of the KDIGO guidelines in 2024 [7]. However, the uptake remains extremely low. Possible explanations include limited clinician familiarity with the drug, its relatively recent reimbursement, and concerns over adverse effects such as hyperkalemia. Structural barriers, such as lack of clinical decision support tools, may also contribute to underutilization.

The low rate of potassium binder use observed in our cohort can be seen as a gap in the management of hyperkalemia in routine clinical practice. Nevertheless, hyperkalemia history was relatively low in the tested population. Nowadays, effective therapies to control potassium levels and mitigate associated risks are available [14,15]. In cardiorenal diseases, potassium binders allow the continuation or even uptitration of hyperkalemia-inducing medications (RASi, MRA), resulting in beneficial long-term outcomes [16,17]. Our findings suggest that, even though hyperkalemia is not so common among CKD patients in everyday cardiology practice, there is a lack of potassium binder uptake. Possible reasons may include concerns about cost, side effects, or a perceived lack of urgency in addressing mild to moderate hyperkalemia [18]. Further research is warranted to explore barriers to the use of potassium binders and to develop strategies that promote their appropriate integration into care.

Notably, nephrology consultation was not associated with a higher likelihood of patients being on SGLT2 inhibitors prior to admission. This is unexpected given the central role of nephrologists in CKD management and their presumed familiarity with renoprotective therapies. One possible explanation is that in the Greek healthcare system, outpatient care for CKD is often fragmented, and nephrology involvement may occur late or only after significant disease progression. Furthermore, referrals to nephrology may focus on dialysis planning or lab interpretation rather than long-term medication optimization. It is also plausible that nephrologists were hesitant to initiate SGLT2 inhibitors prior to their reimbursement for non-diabetic CKD in February 2023. These findings underscore the need to clarify the outpatient roles of nephrologists in prescribing foundational CKD therapies and support further qualitative work to explore referral patterns, decision-making, and barriers to initiation in the ambulatory setting.

Managing physician inertia and related barriers in CKD therapy adoption requires targeted strategies to increase awareness, education, and ease of access to these treatments. Educational interventions, such as workshops, guidelines refreshers, and continuing medical education modules, are essential to ensure that clinicians remain updated on evolving evidence for CKD management. Integrating decision support tools within electronic health records could provide real-time prompts for prescribing SGLT2i and finerenone in eligible patients, supporting evidence-based decisions at the point of care. Moreover, fostering a collaborative approach through regular team meetings and case discussions among nephrologists, cardiologists, and general practitioners can encourage consistent application of guideline-directed therapies. Patient engagement strategies, such as shared decision-making and educational sessions, can also play a role, empowering patients to discuss and advocate for renoprotective treatments with their healthcare providers.

Polypharmacy can be another contributing factor to the non-ideal prescription rates, either by means of multiple pills that often are not welcomed by patients, or its cost, which can be considerably high for specific age groups. However, CKD medication prescription in Greece is accompanied by full reimbursement, so the cost cannot be seen as a potential barrier for the prescription of these treatments. Current research is focused on fixed combination therapies targeting even patients with more advanced kidney disease. Such combined therapies could improve prescription rates in the future.

To our knowledge, there is a paucity of data regarding the implementation of renoprotective pharmacotherapy in patients with CKD, constituting a major strength of the present study. Moreover, despite the cross-sectional design of the study, the fact that HECMOS was performed twice within a 2-year period, during which significant changes in guideline recommendations and drug reimbursement occurred, gave us the possibility to detect longitudinal trends in drug prescription. However, we acknowledge certain limitations. As this study employed repeated cross-sectional snapshots without patient-level follow-up, temporal trends in therapy use can be described, but causal inferences regarding prescribing behavior or outcomes cannot be made. Τhe study’s reliance on self-reported and hospital-recorded data may have introduced recall or documentation bias, particularly if medication usage or patient comorbidities were under- or over-reported. Importantly, as we did not have access to previous renal function assessments, we could not determine potential variations in kidney function that would have precluded SGLT2i use. However, the proportion of such patients is considered low and could not have influenced the overall findings. In addition, we were unable to capture reasons for non-prescription of SGLT2i and finerenone, such as specific physician concerns, patient preferences, or barriers related to healthcare access and cost, which may further contextualize our findings. The concept of physician inertia, while plausible, cannot be confirmed by this study due to the absence of behavioral data. Collecting qualitative data through physician surveys, patient interviews, or prescribing audits could provide valuable insights into these barriers. Third, although we accounted for hospital-level clustering using multilevel modeling, the observed ICCs indicate that a significant proportion of prescribing variation was attributable to institutional factors. However, we did not capture specific hospital-level characteristics (e.g., formulary restrictions, local prescribing policies, or presence of clinical decision support systems) that could further explain this variability. As such, unmeasured institutional confounding may persist and should be explored in future studies using site-level data or qualitative methods. Fourth, the study population consisted exclusively of patients admitted to cardiology departments across Greece and may not be generalizable to primary care, nephrology outpatient clinics, or healthcare systems in other countries. Differences in healthcare infrastructure, physician specialty, and national guidelines may significantly influence prescribing behavior. However, we focused on prescription patterns prior to admission in an attempt to reflect real-world utilization trends. Finally, we did not account for potential differences in regional or institutional practices that could influence prescribing patterns, nor did we assess the impact of these therapies on clinical outcomes among the patients studied.

## 5. Conclusions

In these nationwide cross-sectional analyses, we observed a doubling in SGLT2i use among cardiology inpatients with CKD, yet overall utilization of renoprotective therapies—especially finerenone—remains suboptimal. Physician- and system-level barriers appear to hinder optimal uptake. Tailored implementation strategies, such as educational programs, decision-support tools, and multidisciplinary collaboration, are needed to bridge this gap and align clinical practice with updated guideline recommendations.

## Data Availability

The datasets created to support the findings of this manuscript are available upon reasonable request from the corresponding author.

## Supplementary Materials

The following supporting information can be downloaded at: https://www.mdpi.com/article/10.3390/biomedicines13081987/s1, Table S1: Characteristics of the study population according to CKD status. Table S2: Characteristics of patients with CKD according to SGLT2i use. Table S3: Predictors of SGLT2i use prior to index hospitalization in patients with a history of CKD in HECMOS.
