# Supplementary material for: Trends in Utilization of Guideline-Directed Cardiorenal Protective Therapies for Chronic Kidney Disease in Patients with Cardiovascular Morbidity: Real World Data from Two Cross-Sectional Snapshots (HECMOS I and II)
Source: Biomedicines. 2025 Aug 15;13(8):1987. doi: 10.3390/biomedicines13081987 (PMC12383412; doi:10.3390/biomedicines13081987)
Supplement: Supplementary file 1 [file biomedicines-13-01987-s001.zip › Table S3.pdf]

Supplementary Table S3. Predictors of SGLT2i use prior to index hospitalization in patients with a history of CKD in HECMOS.

**HECMOS 1**			
**Parameter**	**OR**	**95% CI**	**p**
Age	0.95	0.90-0.99	0.015
DM	12.01	3.31-45.56	<0.001
History of CHF	5.01	1.05-24.58	0.043
History of ASCVD	1.81	0.74-4.45	0.20
**HECMOS 2**			
**Parameter**	**OR**	**95% CI**	**p**
Age	0.97	0.94-1.00	0.031
Male sex	1.13	0.59-1.19	0.71
DM	1.23	0.69-2.19	0.49
History of CHF	4.10	1.70-9.88	0.002
History of ASCVD	1.75	0.95-3.24	0.057

DM: diabetes mellitus, CHF: chronic heart failure, ASCVD: atherosclerotic cardiovascular disease, CKD: chronic kidney disease, CRS: cardiorenal syndrome
