# Supplementary material for: Trends in Utilization of Guideline-Directed Cardiorenal Protective Therapies for Chronic Kidney Disease in Patients with Cardiovascular Morbidity: Real World Data from Two Cross-Sectional Snapshots (HECMOS I and II)
Source: Biomedicines. 2025 Aug 15;13(8):1987. doi: 10.3390/biomedicines13081987 (PMC12383412; doi:10.3390/biomedicines13081987)
Supplement: Supplementary file 1 [file Table S1.pdf › Table S1.pdf]

Supplementary Table S1. Characteristics of the study population according to CKD status.								
**Parameter**	**HECMOS 1**				**HECMOS 2**			
	**Total (N=923)**	**CKD (N=240)**	**Non-CKD (693)**	**P**	**Total (N=1175)**	**CKD (N=319)**	**Non-CKD (N=856)**	**p**
Age, years	70.2 (14.8)	76.9 (16.9)	67.8 (15.3)	<0.001	71.0 (14.6)	78.1 (10.1)	68.6 (15.0)	<0.001
Male sex, %	68.2	74.9	65.9	0.01	65.2	66.7	64.7	0.53
BMI, kg/m^2	27.8 (4.9)	27.3 (4.7)	28.0 (5.0)	0.07	27.5 (5.1)	27.7 (5.2)	27.3 (5.2)	0.21
**Prior Medical History**								
Hypertension, %	67.4	76.7	64.0	<0.001	69.5	79.1	66.5	<0.001
DM, %	32.8	54.4	25.4	<0.001	31.1	45.8	25.8	<0.001
AF, %	30.5	44.3	25.6	<0.001	34.9	52.2	28.5	<0.001
ASCVD, %	33.8	52.1	27.2	<0.001	26.5	35.5	23.3	<0.001
CHF, %	41.8	76.7	29.4	<0.001	44.5	76.8	32.4	<0.001
**Medications prior to index admission**								
Diuretics, %	45.8	72.3	36.4	<0.001	44.0	74.0	33.4	<0.001
MRA, %	13.1	31.7	13.1	<0.001	19.5	33.9	14.4	<0.001
RASi, %	44.1	42.1	44.8	0.47	42.4	41.7	42.6	0.77
SGLT2i, %	8.0	13.8	6.0	<0.001	19.0	30.4	15.2	<0.001
**CKD-related parameters**								
Nephrologist consultation, %		27.4				38.9		
CRRT, %		7.6				10.9		
Prior CKD-related hospitalization, %		8.9				13.3		

CKD: chronic kidney disease, BMI: body mass index, DM: diabetes mellitus, AF: atrial fibrillation, ASCVD: atherosclerotic cardiovascular disease, CHF: chronic heart failure, MRA: mineralocorticoid receptor antagonist, RASi: renin-angiotensin system inhibitor, SGLT2i: sodium-glucose cotransporter 2 inhibitor, CRRT: chronic renal replacement therapy
Categorical variables are presented as percentages.
Continuous variables are presented as mean (standard deviation)
