# Supplementary material for: Trends in Utilization of Guideline-Directed Cardiorenal Protective Therapies for Chronic Kidney Disease in Patients with Cardiovascular Morbidity: Real World Data from Two Cross-Sectional Snapshots (HECMOS I and II)
Source: Biomedicines. 2025 Aug 15;13(8):1987. doi: 10.3390/biomedicines13081987 (PMC12383412; doi:10.3390/biomedicines13081987)
Supplement: Supplementary file 1 [file Table S2.pdf › Table S2.pdf]

Supplementary Table S2. Characteristics of patients with CKD according to SGLT2i use.						
**Parameter**	**HECMOS 1**			**HECMOS 2**		
	**SGLT2i users (N=33)**	**SGLT2i non-users (N=187)**	**P**	**SGLT2i users (N=91)**	**SGLT2i non-users (N=188)**	**P**
Age, years	73.7 (10.3)	78.0 (10.0)	0.03	76.5 (9.6)	80.0 (9.9)	0.008
Male sex, %	74.2	74.0	0.99	74.2	61.3	0.04
BMI, kg/m^2	27.3 (4.2)	27.5 (4.7)	0.79	27.8 (4.7)	28.0 (5.3)	0.83
**Prior Medical History**						
Hypertension, %	69.7	74.9	0.69	71.1	81.0	0.07
DM, %	90.6	47.5	<0.001	55.7	42.1	0.035
AF, %	48.5	46.2	0.81	58.2	49.5	0.17
ASCVD, %	69.7	49.2	0.03	50.6	27.0	<0.001
CHF, %	93.8	73.1	0.01	92.0	69.2	<0.001
**Medications prior to index admission**						
Diuretics, %	93.8	72.6	0.01	91.2	69.1	<0.001
MRA, %	57.6	28.9	0.001	60.4	24.5	<0.001
RASi/ARNI, %	43.9	39.4	0.63	51.6	52.1	0.94
**CKD-related parameters**						
Nephrologist consultation, %	24.2	23.0	0.88	33.0	33.9	0.81
Prior CKD-related hospitalization, %	5.3	6.1	0.79	8.9	8.2	0.46

CKD: chronic kidney disease, BMI: body mass index, DM: diabetes mellitus, AF: atrial fibrillation, ASCVD: atherosclerotic cardiovascular disease, CHF: chronic heart failure, MRA: mineralocorticoid receptor antagonist, RASi: renin-angiotensin system inhibitor, ARNI: angiotensin receptor/neprilysin inhibitor, SGLT2i: sodium-glucose cotransporter 2 inhibitor, CRRT: chronic renal replacement therapy

Categorical variables are presented as percentages.

Continuous variables are presented as mean (standard deviation)
